# Supplementary material for: Drug-cured experimental Trypanosoma cruzi infections confer long-lasting and cross-strain protection
Source: PLoS Negl Trop Dis. 2020 Apr 17;14(4):e0007717. doi: 10.1371/journal.pntd.0007717 (PMC7190179; doi:10.1371/journal.pntd.0007717)
Supplement: S1 Fig — Vaccinated BALB/c mice (n = 5) (Materials and Methods) were infected i.p. with 103 bioluminescent T. cruzi trypomastigotes and monitored by ex vivo bioluminescence imaging. Organs, harvested 95 days post-challenge, were arranged as indicated. All images use the same log10-scale heat-map with minimum and maximum radiance values as shown. (PPTX) [file pntd.0007717.s001.pptx]

## Slide 1
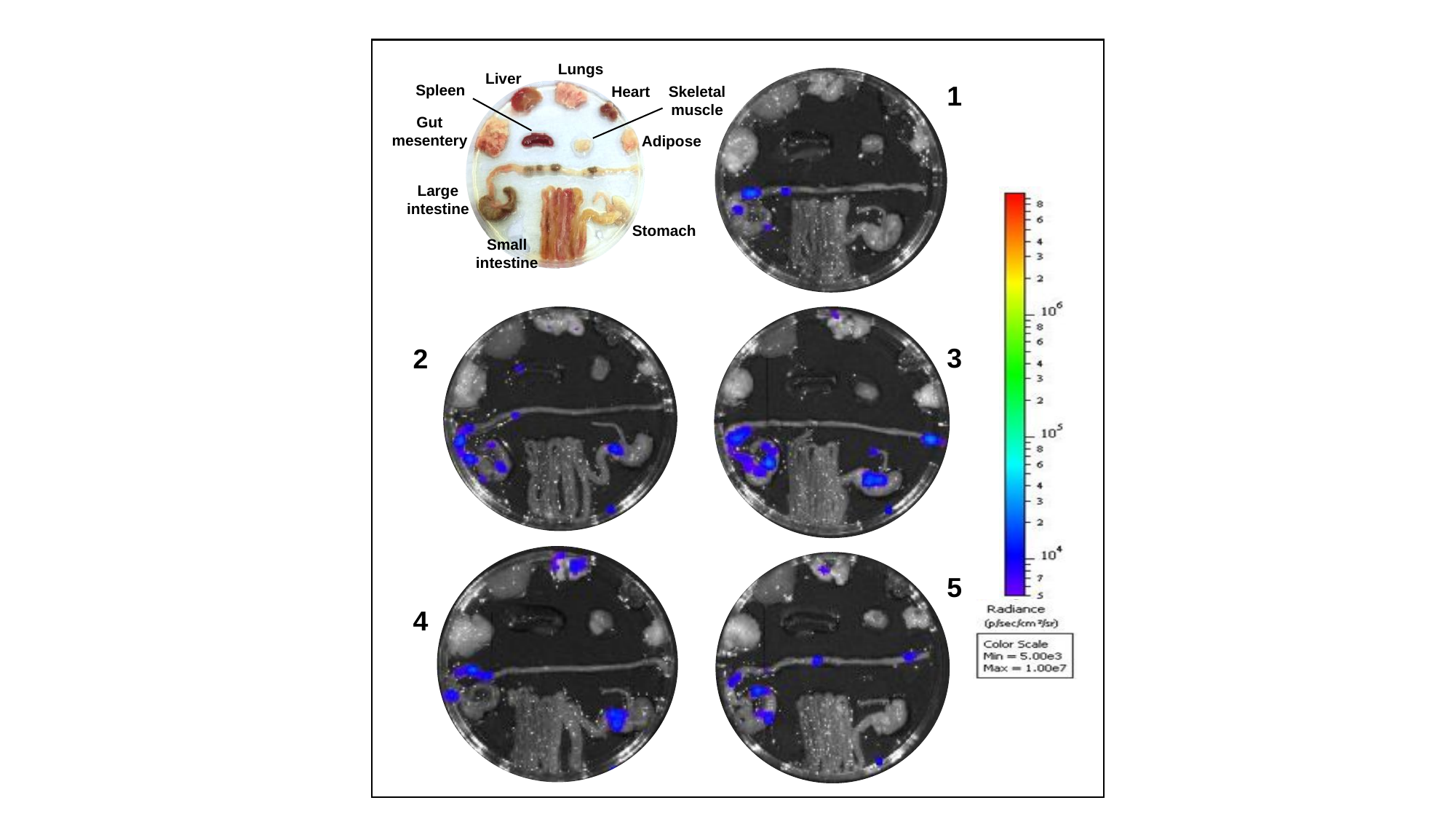

Lungs
Liver
Spleen
Skeletal muscle
Heart
Gut mesentery
Adipose
Large intestine
Stomach
Small intestine
1
3
5
2
4
