# Supplementary material for: Drug-cured experimental Trypanosoma cruzi infections confer long-lasting and cross-strain protection
Source: PLoS Negl Trop Dis. 2020 Apr 17;14(4):e0007717. doi: 10.1371/journal.pntd.0007717 (PMC7190179; doi:10.1371/journal.pntd.0007717)
Supplement: S2 Fig — (A, B) In vivo imaging of BALB/c mice infected with CL Brener (A) and JR (B) strains of T. cruzi. Treatment with benznidazole, 100 mg kg-1 once daily by the oral route for 20 days, was initiated 36 days post-infection. Following cessation of treatment, mice were immunosuppressed with 3 doses of 200 mg kg-1 cyclophosphamide (Materials and Methods). All images use the same log10-scale heat-map with minimum and maximum radiance values indicated. (C and D) Total body bioluminescence (sum of ventral and dorsal images) of CL Brener (C) and JR (D) infected mice. Dashed lines indicate background bioluminescence. All images use the same log10-scale heat-map with minimum and maximum radiance values indicated. (E and F) Ex vivo bioluminescence imaging of organs and carcasses from CL Brener (E) and JR (F) infected mice at the experimental end-point. A minor bioluminescent focus was observed in the adipose tissue of mouse 1 (JR infection). Mouse 2 (CL Brener infection) was euthanised prior to day 89, due to weight loss during immunosuppressive treatment. It was negative by both in vivo and ex vivo imaging. (PPTX) [file pntd.0007717.s002.pptx]

## Slide 1
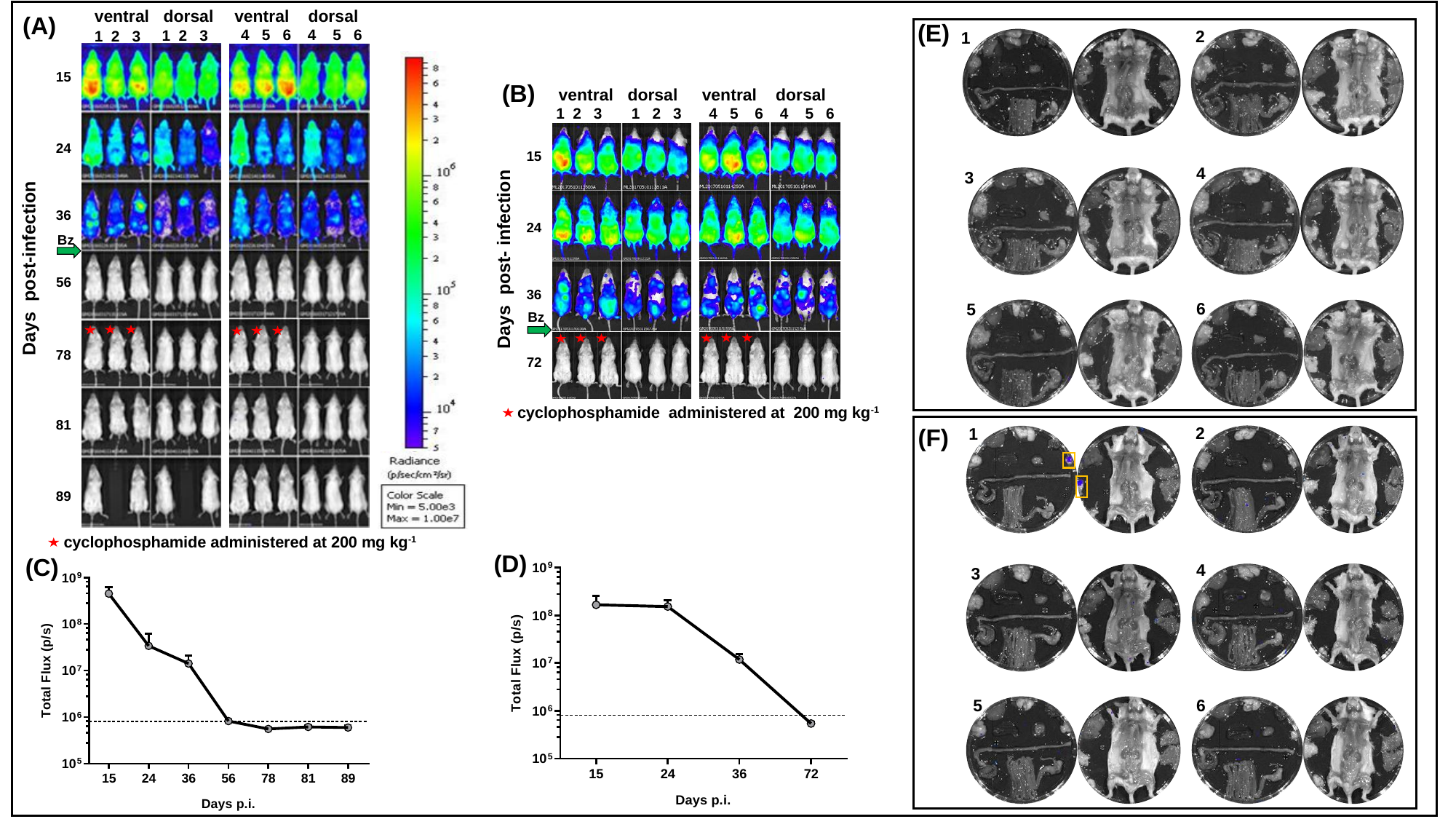

ventral dorsal
ventral dorsal
(A)
 (E)
4 5 6 4 5 6
2
1 2 3
1 2 3
1
15
(B)
ventral dorsal
ventral dorsal
1 2 3 1 2 3
4 5 6 4 5 6
24
15
4
3
36
24
Days post-infection
Bz
Days post- infection
56
36
6
5
Bz
78
72
 cyclophosphamide administered at 200 mg kg-1
81
(F)
2
1
89
cyclophosphamide administered at 200 mg kg-1
(D)
(C)
4
3
5
6
