# Supplementary material for: Drug-cured experimental Trypanosoma cruzi infections confer long-lasting and cross-strain protection
Source: PLoS Negl Trop Dis. 2020 Apr 17;14(4):e0007717. doi: 10.1371/journal.pntd.0007717 (PMC7190179; doi:10.1371/journal.pntd.0007717)
Supplement: S3 Fig — (A) Timeline. BALB/c mice infected i.p. with 103 trypomastigotes (CL Brener strain) were subjected to curative benznidazole treatment (20 days, 100 mg kg-1) initiated 4 days or 36 days post-infection. 23 days after the end of treatment, they were re-infected i.p. After a further 75 days, the mice were immunosuppressed using cyclophosphamide (red stars) (3 x i.p. injections at 200 mg kg-1 over 9 days) and assessed by ex vivo imaging on day 90. (B and C) Ventral and dorsal bioluminescence images from cohorts of 6 mice where treatment was initiated 4 days and 36 days, respectively, after the primary infection. The days post re-infection are indicated (left). All images use the same log10 scale heat-map with minimum and maximum radiance values indicated. (D and E) Ex vivo bioluminescence imaging of organs and carcasses harvested at the experimental end-point (day 90). Yellow rectangles highlight bioluminescent foci. In the 4 day primary infection experiment, only one mouse (#3) was designated as protected, whereas in the 36 day experiment, only one mouse was non-protected (#2). (PPTX) [file pntd.0007717.s003.pptx]

## Slide 1
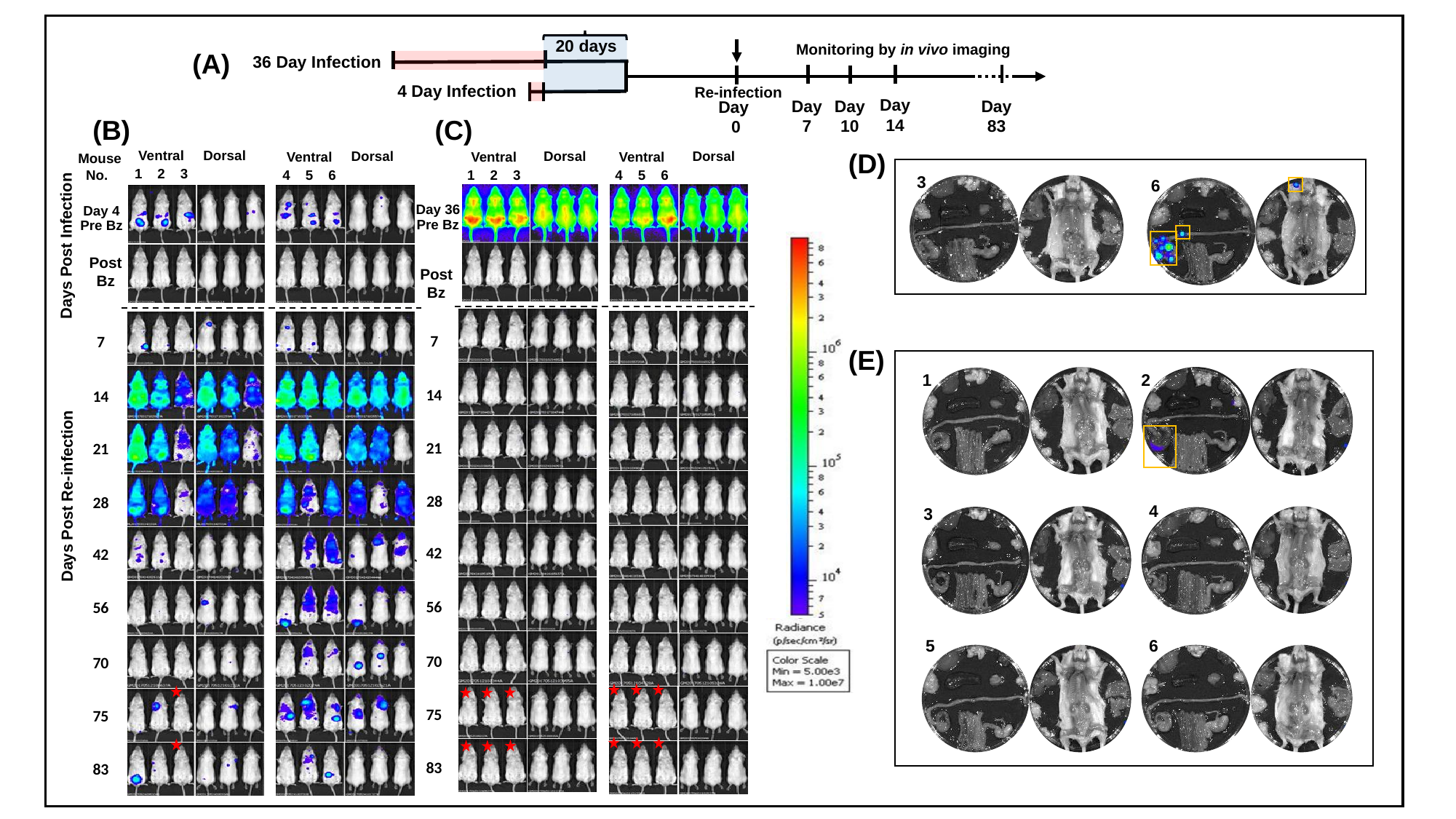

20 days
Monitoring by in vivo imaging
(A)
36 Day Infection
4 Day Infection
Re-infection
Day 14
Day
7
Day 83
Day 10
Day
0
(B) (C)
(D)
(E)
Mouse
 No.
Dorsal
 Ventral
 1 2 3
Dorsal
Dorsal
Dorsal
 Ventral
4 5 6
 Ventral
1 2 3
 Ventral
4 5 6
3
6
Day 36
Pre Bz
Day 4
Pre Bz
Days Post Infection
Days Post Infection
Post Bz
Post Bz
7
7
1
2
14
14
21
21
Days Post Re-infection
28
28
Days Post Re-infection
4
3
42
42
56
56
5
6
70
70
75
75
83
83
